# Cases of Vesico-Vaginal Fistula, Treated by Operation

**Published:** 1851-05

**Authors:** 


Art. X.—*Cases of Vesico-Vaginal Fistula, treated by operation.* By George Hayward, M. D.

We are indebted to Dr. Hayward for a copy of this interesting monograph. It contains the details of nine cases, which were operated on twenty times. The concluding remarks of this contribution to practical medicine will interest many of the readers of this Journal, and we quote them:

Though I have extended this paper to somewhat of an unreasonable length, I hope I shall be excused for adding a few words in order to explain, a little more in detail, the mode I have adopted in doing the operation, and of managing the patients afterwards.

Before the discovery of the anæsthetic powers of ether, I found that the most difficult and painful part of the operation consisted in bringing the bladder down to the os externum. It is now done with comparative ease, and without causing the slightest suffering to the patient. I have administered the ether in the three last operations of this kind, and have been able to bring the bladder down, pare the edges of the fistula, introduce the ligatures and the catheter, and restore the bladder to its place, in twenty minutes; when in all the cases before, in which I did not use it, the same process required an hour, and during the most of that time the patient was suffering severely. Besides, the fistula is sometimes in such a situation, as when it is near the fundus of the bladder, that without this agent, or some similar one, it would be impossible to bring it in view.

The patient being thoroughly etherized, the bladder can be brought down by introducing a large-sized bougie (one made of whalebone, highly polished, is to be preferred) into the urethra, to the very fundus of the bladder, and carrying the other end up to the pubis. In this way the fistula is readily brought in sight. Its edges can be pared with the scissors or a knife, though usually both these instruments are required; and this part of the operation is much facilitated by holding the edges by means of a double hook. In all the cases that I have examined, these edges are thick, hard, and usually of a white color. It is not difficult, therefore, to dissect up the outer covering from the mucous coat of the bladder to the distance of two or three lines. The needles are then to be passed through the outer covering only, and as many stitches must be introduced as may be found necessary to bring the edges of the fistula in close contact.

Since my first operation, I have used a short needle with the eye near the point, made to fit on to a long handle. The instrument, when the two parts are together, looks not much unlike a tenaculum, though not so much curved, and considerably broader near the point.

As soon as the needle is passed through one side of the fistula, it is immediately seized by a forceps, the handle is withdrawn, and the needle is then carried through. It is to be then again fitted to the handle, and carried through to the other side in the same way. As many stitches as may be thought necessary to bring the parts into close contact can in this way be taken with great ease. One thread of each stitch is to be cut off; it is convenient to leave the other, as it enables the operator and patient to know when the ligatures have separated from the bladder.

A large sized female catheter is then to be introduced into the bladder, and secured there by means of a T bandage. The patient should be laid on her side, with the upper part of the body somewhat raised, so as to facilitate the flow of water through the catheter. This should be removed at least once in every twenty-four hours, as it is very likely to be obstructed by mucus, coagula of blood, and occasionally calculous concretions. In three days I think it safe to remove it altogether, but then it should be introduced at least once every three hours, for ten or twelve days more, so as to prevent any accumulation of urine in the bladder, and consequent strain on that organ.

The diet should consist entirely of liquid, mucilaginous food; such as an infusion of slippery elm, gum arabic and water, flaxseed tea, arrowroot, and milk and water. This diet, in my opinion, should be continued till the ligatures come away.

The bowels should be opened by some mild laxative a few hours before the operation; but it is desirable that they should not be moved again till some days after.

I think it best for the patient to use the catheter once or twice a day for several weeks, and at any rate during that time to avoid making any strong efforts to expel the urine by the contraction of the bladder.

It may be proper to add, that I have never had any troublesome hemorrhage from the operation, nor any alarming symptoms after it. In some cases the pain has been severe for two or three days, and once or twice it has run down the limb, apparently in the course of the sciatic nerve. When performed in the way that I have recommended, I believe it to be attended with very little if any danger, as the bladder is not subjected to any considerable degree of violence, nor any part injured to a great extent.

